# Fire needle for recurrent aphthous stomatitis: A protocol for systematic review and network meta-analysis

**DOI:** 10.1097/MD.0000000000028731

**Published:** 2022-02-11

**Authors:** Jun Chen, Xingxin Wang, Kai Wang, Zhihao Liu, Xiaoya Lv, Ming Wang, Shoudan Sun

**Affiliations:** a Shandong University of Traditional Chinese Medicine, Jinan, Shandong, China; b Shandong Provincial Third Hospital, Cheeloo College of Medicine, Shandong University, Jinan, Shandong, China.

**Keywords:** acupuncture, fire needle, protocol, recurrent aphthous stomatitis, systematic review

## Abstract

**Background:**

Recurrent aphthous stomatitis (RAS), are common inflammatory lesions of the oral mucous, usually round or ovoid, circumscribed by erythematous haloes with a yellow-grey floor and mostly painful. The purpose of this study was to evaluate the efficacy and safety of fire needle in the treatment of RAS.

**Methods:**

PubMed, EMBASE, the Cochrane Library, Chinese National Knowledge Infrastructure, Chinese VIP Information, Wanfang Database, and Chinese Biomedical Literature Database were searched by 2 reviewers from the inception until December 2021. The original study that randomized control trials of fire needle for RAS will be selected and is not limited by country or language. In addition, researches in progress, the reference lists and the citation lists of identified publications will be retrieved similarly. Study selection, data extraction, and assessment of the quality will be performed independently by 2 reviewers who have been trained prior to data extraction. A meta-analysis will be conducted if the quantity and quality of the original studies included are satisfactory; otherwise, a descriptive analysis will be conducted. Review Manager V5.4 software will be using for data synthesis and assessment of the risk of bias according to Cochrane Handbook.

**Results:**

This study will provide a comprehensive review of current evidence for the treatment of fire needle on RAS.

**Conclusion:**

The conclusion of this study will provide a judging basis that whether the treatment of RAS with fire needle is effective.

**INPLASY registration number:**

INPLASY2021120118.

## 1 Introduction

Recurrent aphthous stomatitis (RAS), are common inflammatory lesions of the oral mucous, usually round or ovoid, circumscribed by erythematous haloes with a yellow-grey floor and mostly painful.^[1]^ RAS patients usually experience prodromal burning sensations that last from 2 to 48 hours before an ulcer appears. Ulcers are round with well-defined erythematous margins and a shallow ulcerated center covered with yellowish-gray fibrinous pseudomembrane. RAS ulcers usually develop on non-keratinized oral mucosa, with the buccal and labial mucosa being the most common sites, and last approximately 10 to 14 days without scar formation.^[2]^ RAS is often idiopathic but can be associated with gastro-intestinal diseases (i.e., celiac disease, inflammatory bowel diseases), nutritional deficiencies (iron, folates…), immune disorders (HIV infection, neutropenia), and rare syndromes.^[3]^

Approximately 20% of the general population is affected by RAS, but incidence varies from 5% to 50% depending on the ethnic and socioeconomic groups studied.^[4,5]^ The prevalence of RAS is influenced by the population studied, diagnostic criteria, and environmental factors.^[6]^ In children, prevalence of RAS may be as high as 39% and is influenced by the presence of RAS in 1 or both parents.^[7]^ Children with RAS-positive parents have a 90% chance of developing RAS compared with 20% of those with RAS-negative parents.^[4]^ In children of high socioeconomic status, RAS is 5 times more prevalent and represents 50% of oral mucosal lesions in this cohort.^[8,9]^ RAS prevalence was found to be higher (male, 48.3%; female, 57.2%) among professional school students than in the same subjects 12 years later when they had become practicing professionals. This finding led some investigators to theorize that stress during student life is a major factor in RAS, although the differences due to age changes should also be considered. The onset of RAS appears to peak between the ages of 10 and 19 years and becomes less frequent with advancing age, geographic location, or gender.^[10]^ If RAS begins or significantly increases in severity after the third decade and well into adult life (see Table 1), it should increase suspicion that the etiology of the condition maybe attributed to an underlying medical disorder such as hematologic, immunologic, connective tissue disease, or Behçet syndrome.^[2]^

**Table 1:** PubMed search strategy.

Number	Search items
#1	“recurrent aphthous stomatitis”[MeSH]
#2	“Aphthous Stomatitides”[Title/Abstract] OR“Aphthous Stomatitis”[Title/Abstract] OR“Stomatitides, Aphthous”[Title/Abstract] OR“Ulcer, Aphthous”[Title/Abstract]OR“Aphthous Ulcer”[Title/Abstract] OR“Aphthous Ulcers”[Title/Abstract]OR“Ulcers, Aphthous”[Title/Abstract]OR“Aphthae”[Title/Abstract]OR“Canker Sore”[Title/Abstract]OR“Canker Sores”[Title/Abstract]OR“Sore, Canker”[Title/Abstract]OR“Sores, Canker”[Title/Abstract]OR“Periadenitis Mucosa Necrotica Recurrens”[Title/Abstract]
#3	“Cautery”[title/abstract]OR“fire needle”[title/abstract] OR“huo zhen”[title/abstract] OR“fire needle moxibustion”[title/abstract] OR“acupuncture”[title/abstract].
#4	“andomized controlled trial”[Title/Abstract] OR“ randomized”[Title/Abstract]OR“ placebo”[Title/Abstract].
#5	#2 and #3 and #4

Many treatments have been advocated for recurrent aphthous ulceration. These may be based upon antiseptics, antibiotics, corticosteroids, immunosuppressants, anti-rheumatics, anti-inflammatories, hormone therapy, antivirals, colchicine, thalidomide, pentoxifylline, sodium cromoglycate, interferon, hyaluronic acid, helicobacter eradication, zinc, various acids, gastric ulcer treatments, ultrasound, laser, cautery, cryotherapy, bioadhesives, herbal remedies, homeopathy, vitamins, lactobacillus as well as sundry other management strategies and combinations of various medications.^[11–13]^ Systemic treatment may be appropriate for more severe and resistant cases. It should be made clear to the patient that the objective of treatment is symptomatic and that the ulcers cannot be “cured”. The plethora of treatments used for the treatment of oral ulceration is testament to the lack of any single effective treatment. There has not been a systemic (Cochrane) review of oral ulceration published.^[14]^

In China, acupuncture and moxibustion are effective traditional therapeutics and fire needle is an operation method in traditional acupuncture therapy. Fire needle with rapid needle, through the sudden warming point to the body to pass nerve, body temperature stimulation information reaction, so as to strengthen the body's immune response to the disease, promote lesion repair. As a non-drug therapy, fire needle has been reported in some clinical studies that has certain curative effect on RAS. Therefore, the purpose of this study was to summarize the original research on the treatment of RAS with fire needle, so as to evaluate whether the treatment of RAS with fire needle is really effective.

## 2 Methods

### 2.1 Registration

This systematic review will aim to evaluate the effect and safety of fire needle therapy for PCs. Our protocol has been registered on the International Platform of Registered Systematic Review and Meta-Analysis Protocols (INPLASY). The registration number was INPLASY2021120118. All steps of this systematic review will be performed according to the Cochrane Handbook.

### 2.2 Inclusion criteria for this overview

PICOS will be applied, including Population, Intervention, Comparison, Outcome, and Study.

#### 2.2.1 Types of studies

Randomized controlled trials (RCTs) with fire needle as the primary intervention for RAS will be included, and other studies such as case reports, and reviews will be excluded. No restrictions on country but language will be limited on English and Chinese.

#### 2.2.2 Types of participants

Participants diagnosed as PCs by clinicians referring to the New Routine for Diagnosis and Treatment^[15]^ will be included. No restrictions on gender, age, race, etc.

#### 2.2.3 Types of interventions

Without limits on course and dose, we will include studies in which fire needle is the primary intervention and, if necessary, we will include studies in which fire needle is combined with other active treatments versus active treatment alone.

#### 2.2.4 Types of comparisons

The selected randomized controlled trials should testify that the interventions were compared with a control group composed of placebo, sham acupuncture, no treatment, or other active therapies.

#### 2.2.5 Outcomes

Primary outcome: visual analog scale and the healing time

Secondary outcomes: recurrence rate; adverse events incidence caused by fire needle, such as dizziness, vomiting, weariness, etc.

### 2.3 Search methods for study identification

#### 2.3.1 Electronic searches

The 2 authors will independently search English databases (PubMed, Embase, and Web of Science), Chinese databases (CNKI, Wanfang database, CBM, and VIP), and clinical registration platforms (Cochrane Library, Chinese Cochrane Centre's Clinical Trial Registry Platform). The search time is from the establishment of the database to December, 2021. Boolean algorithm is used as search formula to search full-text articles with subject terms and free words. The search formula is as follows: (((((((((((((recurrent aphthous stomatitis [Title/Abstract])OR(Stomatitis, Aphthous[Title/Abstract]))OR (Aphthous Stomatitides

[Title/Abstract])) OR (Aphthous Stomatitis[Title/Abstract]))OR(Stomatitides, Aphthous[Title/Abstract]))OR(Ulcer, Aphthous[Title/Abstract]))OR(Aphthae[Title/A-bstract])) OR (Canker Sore[Title/Abstract])) OR (Canker Sores[Title/Abstract]))OR (Sore, Canker[Title/Abstract])) OR(Sores, Canker[Title/Abstract])) OR (Periadenitis Mucosa Necrotica Recurrens[Title/Abstract])) AND (((((fire needle[Title/Abstract]) OR (Cautery[Title/Abstract])) OR (huo zhen[Title/Abstract])) OR (fire needle moxibustion[Title/Abstract])) OR(acupuncture[Title/Abstract]))) AND (((andomized controlled trial[Title/Abstract]) OR (randomized[Title/Abstract])) OR (placebo[Title/Abstract])).

#### 2.3.2 Searching other resources

The relevant published references and citation list will be retrieved in Web of Science. In addition, the relevant systematic reviews or overview will also be identified for additional relevant studies. Moreover, relevant paper versions of medical journals and journals will be screened to ensure that the original studies that not were included in the electronic databases could be included possibly.

### 2.4 Data collection and analysis

#### 2.4.1 Study selection

**Figure 1:**
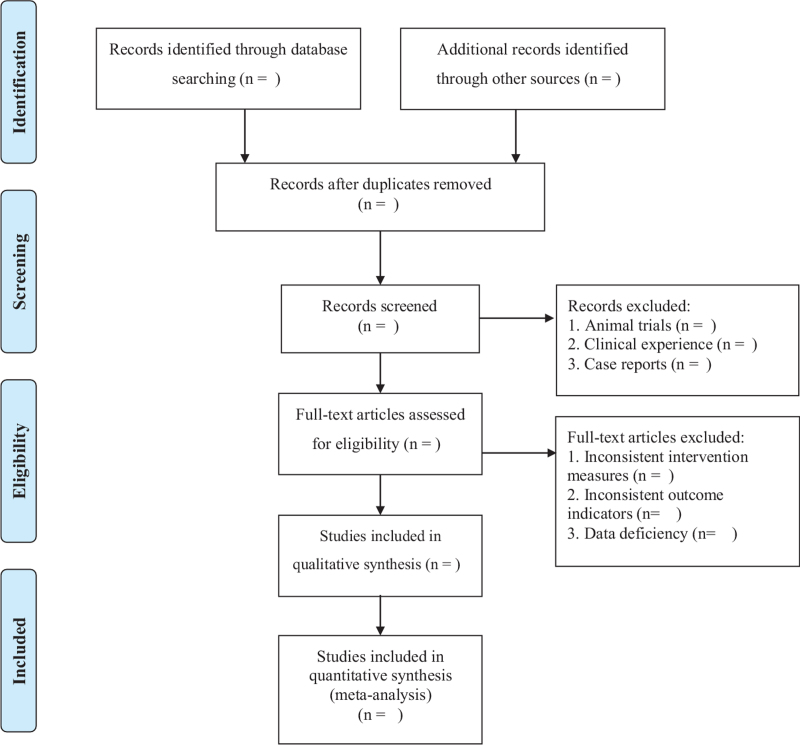
PRISMA flow chart.

All reviewers undergo rigorous training prior to selecting the study. Preliminary screening of the study will be conducted by 2 reviewers independently. After searching, the duplicated studies will be removed initially from the retrieved studies by Endnote (X9) Clarivate Analytics (Beijing, China). And then, 2 independent reviewers will screen titles, abstracts, and keywords of all retrieved studies for candidates according to the inclusion and exclusion criteria, we will obtain the full text of all possibly relevant studies. Excluded studies will be recorded with explanations. If it is uncertain whether to adopt because of the lack of information, Jun Chen will try to contact authors of the original reports to obtain the lost information. During the procedure, disagreements will be resolved by discussion or consensus with the third reviewer. Study selection will be performed in accordance with the Preferred Reporting Items for Systematic Reviews and Meta-Analyses flowchart (Fig. 1).

#### 2.4.2 Data extraction and management

All candidate articles were evaluated and extracted by 2 independent authors. If disagreement occurred, 2 authors discussed and arrived at consensus with a third author. Information from each article will be recorded based on the following table in an Excel document; which includes first author, year of publication, country of publication, study design, total number of cases and gender, follow-ups, treatment strategy, control strategy, etc (Table 1).

#### 2.4.3 Assessment of risk of bias

To systematically evaluate the quality of each of the studies that are finally included. Two reviewers will assess the risk of bias for each included study according to the Cochrane handbook. It will eventually be rated on 3 levels (“high risk of bias”, “medium risk of bias”, and “low risk of bias”).^[16]^ The specific evaluation items include the following 7 aspects: generation of random sequence, allocation concealment, blindness of participants and personnel, blindness of outcome assessment, incomplete outcome data, selective reporting, and other bias.

#### 2.4.4 Measures of treatment effect

Review Manager (RevMan V 5.4, The Nordic Cochrane Centre, The Cochrane Collaboration, Copenhagen, Denmark) will be used for data analysis and quantitative data synthesis. We will use the weight mean difference and 95% confidence interval to measure the continuous variables, while the results of dichotomous variables will use risk ratio and its 95% confidence interval.

#### 2.4.5 Dealing with missing data

If the specific information we need to collect are not reported, the reviewer will attempt to contact the original author for relevant information by telephone or e-mail. If the required information is not available, it will be explained in the article. Then, the missing data will be assumed to be “missing at random” and “missing not at random” according to the Cochrane Handbook.^[17]^ For the data missing at random, the analysis will rely on existing data, while we will fill the missing data with replacement values and make a sensitivity analysis to examine the potential impact of missing information, if necessary.

#### 2.4.6 Assessment of heterogeneity

Heterogeneity refers to the difference between studies in the systematic review,^[18]^ and the value of I^2^ represents the heterogeneity after data synthesis. We will use I^2^ to assess statistical heterogeneity between trials. If the I^2^ < 50%, that indicates slight or no heterogeneity in the evidence of the combined results, while I^2^ ≥ 50%, it means studies with high heterogeneity. The fixed effects model will be adopted when the *P* > .1 and I^2^ <50%, while apply the random effect if *P* < .1 and I^2^ ≥ 50%.

#### 2.4.7 Assessment of reporting bias

An assessment of the reported bias will be presented in the form of a funnel plot. If the points on both sides of the funnel plot are scattered and asymmetric, it is considered that there is a report bias and the reliability of this study is low. On the contrary, if the point distribution on both sides of the funnel plot is symmetrical, we believe that there is no or very low reporting bias, and the results of this study are reliable.

#### 2.4.8 Data synthesis and subgroup analysis

All analysis will be done through RevMan 5.4. According to heterogeneity assessment, mean difference or relative risk were calculated using fixed or random effects models. In addition, if the I^2^ obtained after data consolidation is greater than 50% and the *P* value is less than .1, sensitivity or subgroup analysis will be performed to exclude the source of heterogeneity. If the included original research data are insufficient for quantitative analysis, the review will only represent and summarize the evidence.

#### 2.4.9 Sensitivity analysis

If the results show significant heterogeneity and the number of included studies is sufficient, sensitivity analysis will be performed to identify the quality and robustness of the meta-analysis result, which includes assessing the impact of sample size, methodological elements and the characteristic of research and missing data.

#### 2.4.10 Grading the quality of evidence

The quality of evidence will be evaluated using the Grading of Recommendations Assessment, Development, and Evaluation.^[19]^ The quality of evidences will be rated on 4 levels (high, medium, low, or very low). Two reviewers will conduct the assessment process separately and describe in detail the reasons for downgraded or upgraded outcomes affecting the quality of evidence to guarantee the reliability and transparency of results.

## 3 Discussion

RAS is the most common ulcerative disease affecting the oral mucosa. It occurs mostly in healthy individuals and has atypical clinical presentation in immunocompromised individuals. The etiology of RAS is still unknown, but several local, systemic, immunologic, genetic, allergic, nutritional, and microbial factors, as well as immunosuppressive drugs, have been proposed as causative agents. Clinical management of RAS is based on severity of symptoms, frequency, size, and number of lesions using topical and systemic therapies. The goals of therapy are to decrease pain and ulcer size, promote healing, and decrease frequency of recurrence. As a result, many patients are looking for easier and less harmful alternatives.

As an alternative therapy for external therapy, the fire needle has a history of nearly 3 thousand years in China. It can relieve pain, improve the blood circulation, stimulate metabolism of local tissue.^[20]^ In recent years, a certain amount of studies conducted in China have shown that compared with vitamin B2 and intramuscular injection transfer factor, fire needle has a higher cure rate for the treatment of RAS.

However, the efficacy of fire needles in treating RAS has been controversial due to the lack of evidence-based medicine, and some studies have reported that acupuncture may be a placebo effect. To date, there is no reliable comprehensive review of the treatment of RAS with fire needle. We conducted this study to assess the efficacy of fire needles in the treatment of RAS and to provide clinical staff with a reliable treatment regimen. In addition, through this study, it is believed that more and higher quality original studies will be designed and carried out to provide more accurate guidance for the treatment of RAS.

## Author contributions

All authors have read and approved the publication of the protocol.

**Conceptualization:** Jun Chen, Xingxin Wang, Shoudan Sun.

**Data curation:** Xingxin Wang, Kai Wang, Zhihao Liu, Xiaoya Lv, Ming Wang.

**Formal analysis:** Jun Chen, Kai Wang.

**Methodology:** Jun Chen, Shoudan Sun.

**Software:** Zhihao Liu, Xiaoya Lv.

**Supervision:** Jun Chen, Shoudan Sun.

**Writing – original draft:** Jun Chen.

**Writing – review & editing:** Jun Chen, Shoudan Sun.
